# An Extended Model of the Theory of Planned Behavior: An Empirical Study of Entrepreneurial Intention and Entrepreneurial Behavior in College Students

**DOI:** 10.3389/fpsyg.2022.627818

**Published:** 2022-01-25

**Authors:** Duan Lihua

**Affiliations:** College of Innovation and Entrepreneurship, Anhui Technical College of Mechanical and Electrical Engineering, Wuhu, China

**Keywords:** the theory of planned behavior, entrepreneurial intention (EI), entrepreneurial behavior, extended model, entrepreneurial situational factors, entrepreneurial implementation intention

## Abstract

Currently, there are two bottleneck problems in the research of college students’ entrepreneurial intention and entrepreneurial behavior: lack of comprehensive and systematic theoretical framework and empirical analysis to reveal the role path that affects entrepreneurial intention, and most studies ignore the gap between entrepreneurial intention and behavior. Based on the literature review, this study adopted the Theory of Planned Behavior as the theoretical framework introduced entrepreneurial situational factors and entrepreneurial implementation intention, and constructed a two-step extended entrepreneurial intention–behavior model. The structural equation was constructed using AMOS24.0 to empirically analyze the antecedent variables of college students’ entrepreneurial intention and the factors influencing entrepreneurial behavior. The empirical results showed that expected material possessions, expected social reputation, expected self-evaluation, mission and responsibility, and career development are the antecedent variables of entrepreneurial attitude. Support from families and friends, college teachers’ views, and the role models are antecedent variables of entrepreneurial subjective norms. Professional ability, entrepreneurial ability, entrepreneurial experiences, and personality traits are the antecedent variables of entrepreneurial perceived behavior control. In the formation stage of college students’ entrepreneurial intention, attitude, subjective norms, perceived behavior control, and entrepreneurial situational factors have significant impacts on the formation of college students’ entrepreneurial intention, while entrepreneurial intention, perceived behavior control, and entrepreneurial situational factors have significant impacts on the transformation phase of entrepreneurial behavior. Entrepreneurial implementation intention plays an intervening role between entrepreneurial intention and behavior of college students.

## Introduction

College students are the main force behind innovation and entrepreneurship that promotes economic development (Ever Grande Research Institute, 2020). The number of college graduates pursuing entrepreneurship and their quality, exert a direct impact on the economic growth of a country. According to the MyCOS Chinese College Graduates Employment Report, the percentage of college graduates who were self-employed was 3.0, 3.0, 2.9, 2.7, and 2.8%, for the past 5 years till 2019, respectively (MyCOS Educational Research Company, 2020). However, according to the Chinese College Graduates Employment Report, over 75% of college students showed entrepreneurial intention during undergraduate years (You, 2020). Unfortunately, these undergraduates are not always able to follow their own will, which means that the possibility of realization of their entrepreneurial intention is relatively low, in spite of their willingness to take risks. The actual occurrence of entrepreneurial behavior is the key issue. It is particularly important to study the transformation process from entrepreneurial intention to entrepreneurial behavior of college students.

Currently, theorizing and modeling are commonly used in the researches on entrepreneurial intention and behavior. Among the numerous applied theories, the Theory of Planned Behavior (Ajzen, 1985; Wu, 2017) is well recognized and widely utilized for entrepreneurial intention. However, the research results are relatively scattered, and there is still a lack of comprehensive and systematic theoretical framework and empirical analysis to reveal the path of influence on entrepreneurial intention, as well as the internal relationship among various influencing factors. The famous Theory of Planned Behavior cannot fully explain why college students show strong entrepreneurial intention but do not engage in entrepreneurial activities.

Therefore, keeping the characteristics of college students in mind, introduced entrepreneurial situational factors and entrepreneurial implementation intention, this study established an entrepreneurial intention –behavior two-step extended model based on the Theory of Planned Behavior framework and literature review. The study makes the hypothesis that entrepreneurial situational factors and entrepreneurial implementation intention are the link between entrepreneurial intention and actual behavior and empirically analyzed the factors influencing the formation of entrepreneurial intention, such as antecedent variables, the path of action. Based on the established model, suggestions were made to improve college students’ entrepreneurial intention and behavior.

### Theoretical Foundation

#### Theory of Planned Behavior

**FIGURE 1:**
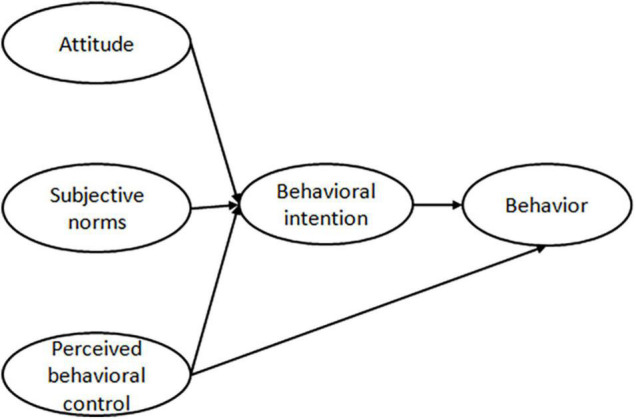
The framework of the Theory of Planned Behavior proposed by Icek Ajzen.

The concept of the Theory of Planned Behavior was proposed by Ajzen (1985). The theory states that an individual’s behavior is influenced directly by behavioral intention and perceived behavioral control. The behavioral intention is shaped by the attitude, perceived behavioral control and subjective norms (Jing et al., 2016; Figure 1). Behavior is a process driven by complex economic, psychological factors, and other decision-making processes. Behavioral intention refers to the possibility of the subject to perform a given behavior. Attitude reflects the subject’s expectation and evaluation of the results of a given behavior. Subjective norms refer to the expectation and attitude of the significant other or group of people toward the subject. Perceived behavior refers to the degree of control and the difficulty of performing a given behavior. The basic the Theory of Planned Behavior framework is mainly applied to study the impact of attitude, subjective norms, and perceived behavioral control on behavioral intention, on the basis of the fact that the subject makes conscious decisions and plans.

#### Entrepreneurial Situational Factors and Entrepreneurial Implementation Intentions

Spilling was the first to clearly put forward the concept of entrepreneurial situational factors, but it was not clearly distinguished from the concept of entrepreneurial environment (Spilling, 1996). Austin et al. (2006) believe that entrepreneurial situational factors is not controlled by entrepreneurs, but will affect the success or failure of entrepreneurship, including macroeconomic, socio-political and regulatory structures. Welter redefined the concept of context related to entrepreneurship on the basis of existing context research, pointing out that situational factors is an environment, situation, condition and situation that is independent of phenomena and can promote or hinder (Welter, 2011).

Intention is a direct factor that affects the realization of specific goals and behaviors in the Theory of Planned Behavior. The point is that moving from intention to behavior is a long-term process that requires individuals to solve a series of execution problems. Individuals may delay behavior due to some factors, or even do not take specific actions after the emergence of intention, resulting in the bottleneck problem of intention to behavior. Therefore, it is particularly important to study the intermediate mechanism and influence of intention to behavior process. Gollwitzer proposed another key factor affecting goal realization through the research on the process from intention to behavior, namely “implementation intentions,” which means to connect specific situational cues with corresponding goal-oriented behavioral responses and determine when, where and how to respond to achieve goals. Entrepreneurial implementation intention is a kind of specific goal intention. The relevant entrepreneurial situational factors can trigger the behavior related to goal intention, and the process is direct, efficient and unconscious (Gollwitzer, 1999). Tatarko and Schmidt (2015) proved that implementation intention is a intervening variable between behavioral intention and behavior and can promote the transformation of intention into behavior.

For college students who are often affected by entrepreneurial situational factors, their behavior of participating in entrepreneurial activities is not the result of conscious decision, but may just be driven by a habit or encounter a specific situation consistent with participating in entrepreneurial activities. But the Theory of Planned Behavior model does not explain this. At present, there are few studies that include both entrepreneurial situational factors and entrepreneurial implementation intention variables in the Theory of Planned Behavior model to explain entrepreneurial activity intention or behavior. According to the Theory of Planned Behavior model, an individual’s behavior is established based on subjective and conscious choices. However, studies have shown that an individual’s unconscious and spontaneous cognitive processes, under certain circumstances, such as situational factors and implementation intentions, also affect intention and actions. Situational factors are influencers, which act upon an individual in the form of environmental cues. College students may develop certain behavioral habits when they attend innovation and entrepreneurship courses offered by colleges or communities and participate in related activities. Such behavioral habits, which are not the result of an individual’s conscious decision, may trigger entrepreneurial intention and gradually elicit entrepreneurial actions in a given situation. An implementation intention is an effective self-regulatory strategy including plan formulation, plan implementation, and implementation effect. Individuals with strong implementation intentions will associate their behaviors with their goals under a given situation. Thus, implementation intention helps to bridge the gap between intention and behavior. Integrating situational factors and implementation intention into the Theory of Planned Behavior model introduces more factors which exert a potential influence on intention and actions, and helps to investigate how subject’s subconsciousness affects the formation of intention and actions. Such an extended the Theory of Planned Behavior model has been validated in studies on travel (Jing et al., 2016) and adolescent sports (Wang and Zheng, 2020).

#### Research Background

Currently, some researchers have explored the determinants of entrepreneurial intention and behavior among college students using the Theory of Planned Behavior -based theoretical or empirical methods. As for the research on the personality traits of entrepreneurs, scholars focus on the “natural” energy, intuition and perseverance of entrepreneurs, as well as the psychological factors such as the needs, attitudes and values of entrepreneurs. Cunningham, J.B. and Lischeron, J. found that entrepreneurs with unique personality traits can promote them to form a certain entrepreneurial intention, on this basis, drive them to take entrepreneurial behavior (Cunningham and Lischeron, 1991). In the research of early entrepreneurial intention, the role of the “Big Five Personality Traits,” risk taking tendency, innovation, locus of control, optimism and other characteristics has been discussed to varying degrees (Liñán and Fayolle, 2015; Donaldson, 2019). Empirical studies have revealed that the “Big Five Personality Traits,” risk-taking tendency, innovation, locus of control and optimism simultaneously have significant positive effects on entrepreneurial intention (Ozaralli and Rivenburgh, 2016). The “Big Five Personality Traits” significantly affected entrepreneurial intention, and the degree of influence on entrepreneurial intention was in the order of openness, emotionality, agreeableness, extroversion and conscientiousness (Zhao et al., 2010; Yang, 2019). In terms of the relationship between personal values and entrepreneurial intention, Saeid and Salman (2020) show that college students in developing countries play an important role in the formation of personal values and entrepreneurial intention. This study shows that educating potential entrepreneurs and educators to inspire personal value can help foster the willingness of potential entrepreneurs to start their own businesses (Saeid and Salman, 2020). In terms of the influence of the power of role models on entrepreneurial intention, Abbasianchavari and Moritz (2020) have found that entrepreneurial role model can enhance entrepreneurs’ entrepreneurial intention and behavior, especially in the early stage of entrepreneurship. The research on the influence of situational factors on entrepreneurial intention holds that the entrepreneurial self-efficacy of individuals who have received entrepreneurial education has a greater influence on entrepreneurial outcome expectation and entrepreneurial behavior tendency than those who have not received entrepreneurial education (Nowiñski et al., 2019; Liu et al., 2020). Study on the temporal stability of entrepreneurial intention and link to behavior, Salo et al. (2020) suggest that entrepreneurial intention is a stable construct over time. High and low levels of entrepreneurial intention remain quite stable. Gender and role models are significant factors in predicting entrepreneurial behavior (Salo et al., 2020).

In China, the application of the Theory of Planned Behavior in entrepreneurial intention and entrepreneurial behavior has also been studied to some extent. Although Fishbein and Ajzen (2010) believe that the entrepreneurial situational factors can only indirectly affect the entrepreneurial intention and entrepreneurial behavior, some scholars of China have pointed out through empirical studies that under the background of Chinese characteristics, entrepreneurship policies, social atmosphere and entrepreneurship education have a direct and huge impact on the entrepreneurial intention and entrepreneurial behavior of college students. An empirical analysis reported by Li (2008) demonstrated that the Theory of Planned Behavior model is suitable for the research of entrepreneurial intention in Chinese college students. They also pointed out that the influence of subjective norms on the entrepreneurial intention of college students in China was higher than that in western countries (Li, 2008; Tang et al., 2015). Liu (2017) introduced entrepreneurial education into the basic the Theory of Planned Behavior framework, constructed a model to investigate the effect of entrepreneurial education on the entrepreneurial intention of college students, and found that entrepreneurial education was the most critical antecedent factor affecting entrepreneurial intention (Qi and Liu, 2010). Hu (2014) conducted an empirical study and demonstrated that the individual’s background and personality traits have a positive effect on the attitude; the attitude and external environment have a positive influence on the entrepreneurial propensity. In his research on entrepreneurial executive intention, Sun Yang believes that individuals with executive intention pay more attention to situational cues related to the behavior of realizing goals, and the more specific the implementation intention is, the more effective it is, and the stronger the sense of control over behaviors is (Sun, 2015).

Literature review shows that although academic circles pay more and more attention to college students’ entrepreneurial intention and entrepreneurial behavior, the existing two problems still restrict the development of college students’ entrepreneurial intention and behavior research. First of all, the Theory of Planned Behavior is well recognized and widely utilized for entrepreneurial intention. However, the research results are relatively scattered, and there is still a lack of comprehensive and systematic theoretical framework and empirical analysis to reveal the path of influence on entrepreneurial intention, as well as the internal relationship among various influencing factors. Secondly, regarding the transformation from entrepreneurial intention to behavior, most studies ignore the gap between entrepreneurial intention and behavior, and few studies discuss the factors influencing the transformation from entrepreneurial intention to behavior of college students. The famous Theory of Planned Behavior cannot fully explain why college students show strong entrepreneurial intention but do not engage in entrepreneurial activities.

College students have unique entrepreneurial situational factors including society, government and universities, and they have the advantage of guiding students to implement entrepreneurial intention under such entrepreneurial situational factors in higher education institutions. Strictly speaking, their entrepreneurial behavior, which might be slightly different from that of their peers, who have been in the workforce, is still a particular behavior that is planned or that with a specific purpose (Su and Zhao, 2019). In the process of transformation from entrepreneurial intention to behavior, entrepreneurial situational factors and entrepreneurial implementation intention play a role need further exploration by researchers. To facilitate innovation and entrepreneurship in college students, it is of important to clarify the influencing factors of college students’ entrepreneurial behavior transformation to promote college students’ innovation and entrepreneurship. Therefore, to facilitate innovation and entrepreneurship in college students, it is of important to clarify the influencing factors of college students’ entrepreneurial behavior transformation to promote college students’ innovation and entrepreneurship.

### Study Hypotheses and Objectives

#### Entrepreneurial Attitude and Entrepreneurial Intention

According to the application study of the Theory of Planned Behavior in entrepreneurial intention, the expected results of starting a business and the perceived utility of such expected results will affect the attitude (Wang et al., 2020). The expected results of pursuing entrepreneurship included expectations on wealth, social reputation, and self-evaluation, *per se*, the college students think that becoming an entrepreneur would bring them various impacts and expectations, such as material, spiritual and value aspects. The perceived utility of entrepreneurship outcome includes the perceived responsibility mission and the perceived utility of career development. During the process of entrepreneurship, graduates contribute to the development of the state and society, as well as to their own career development. The satisfaction and sense of value which comes from the process is referred to as perceived utility of responsibility and mission, and the perceived utility of career development, respectively.

#### Entrepreneurial Subjective Norms and Entrepreneurial Intention

Ajzen analyzed the literature and found that the subjective norms aren’t strong interpreters of intention (Ajzen, 1991). However, Li (2008) suggested that the group variable that has the most influence on the subject in entrepreneurship research should be selected. His empirical study found that entrepreneurial subjective norms have a more significant impact on the entrepreneurial intention of Chinese graduates than that of foreign students (Li, 2008). Krueger et al. (2000), also proved that subjective norms have a significant impact on intentions. Therefore, for testing the formulated hypotheses, the three important reference groups selected for the college students’ entrepreneurial intention—behavior extended model were relatives and friends, college mentors and role models.

#### Entrepreneurial Perceived Behavior Control and Entrepreneurial Intention

Perceived behavior control is affected by human capital, which includes the subject’s professional ability, entrepreneurial ability, experience and personality traits. Professional ability is measured by college students’ professional knowledge and skills. The entrepreneurial ability refers to the knowledge and skills necessary for entrepreneurship, such as management, negotiation, marketing, finance, and law. Practical experience refers to the individual’s experience in a particular industry, entrepreneurship, or commercial activities. The personality traits mean that the student has a mature way of thinking, stays emotionally and behaviorally stable in the entrepreneurial situational factors, is independent, willing to take risks and is innovative.

#### Entrepreneurial Situational Factors, Entrepreneurial Intention and Entrepreneurial Behavior

Entrepreneurial situational factors refer to a combination of many situational factors that can influence college students’ decision to start a business. Based on previous studies, the entrepreneurial situational factors are categorized into: entrepreneurial environment; policies; and education and training. The entrepreneurial environment and policy are the situational factors outside of the college while entrepreneurial education and training are considered as internal factors offered within the college, for e.g., relevant courses, competitions, business incubator bases, etc. Under the influence of the entire social entrepreneurial atmosphere, active participation in the innovation and entrepreneurship courses and other entrepreneurial activities might inspire entrepreneurial intention and eventually promote actual entrepreneurial behavior in college students.

#### Entrepreneurial Implementation Intention, Entrepreneurial Intention, and Entrepreneurial Behavior

According to empirical research in the field of international psychology, implementation intention, as an influence factor for intention and behavior boosts behavioral willingness and promotes the transition from intention to actual behavior (Brickell et al., 2006). Implementation is an effective self-regulatory strategy that bridges a future situation and goal-directed behavior *via* planning that includes plan formulation, plan implementation, and implementation effect. Such planning specifies when, where and how the subject performs a specific behavior to realize the goal. Meanwhile, such planning helps the subject develop behavioral habits unconsciously, promotes entrepreneurial initiative, and becomes the link between intention and actual behavior. Implementation intention to perform makes a strong correlation between the expected situation and the goal-directed behavior, establishing a intervening role between intention and actual behavior (Gollwitzer, 1999).

**FIGURE 2:**
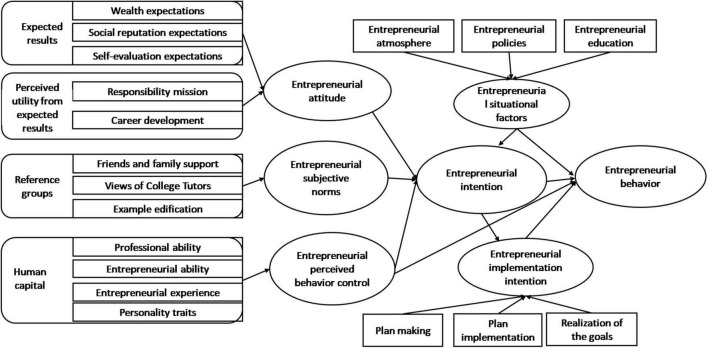
The College students’ entrepreneurial intention—behavior extended model.

Based on the theoretical analysis, this study introduced entrepreneurial situational factors and entrepreneurial implementation intention into the Theory of Planned Behavior framework, established an entrepreneurial intention—behavior two-step extended model. As shown in Figure 2, this study identified the antecedent factors of entrepreneurial intention formation, conducted path analysis using the established model and analyzed the factors that influenced the transformation of entrepreneurial behavior (Table 1).

**TABLE 1:** Hypotheses of college students’ entrepreneurial intention—behavior extended model.

Number	Hypothesis
H1	EA1→EA:Attaining material goals regarding entrepreneurship have a significant positive effect on the entrepreneurial attitude.
H2	EA2→EA:Social reputation expectations regarding entrepreneurship have a significant positive effect on the entrepreneurial attitude.
H3	EA3→EA:Expectation for an individual’s self-evaluation regarding entrepreneurship has a significant positive effect on the entrepreneurial attitude.
H4	EA4→EA:Social responsibility regarding entrepreneurship has a significant positive effect on the entrepreneurial attitude.
H5	EA5→ Career development regarding entrepreneurship has a significant positive effect on the entrepreneurial attitude.
H6	ESN1→ESN:Support from relatives and friends regarding entrepreneurship has a significant positive effect on the entrepreneurial subjective norms.
H7	ESN2→ESN:College teachers’ view regarding entrepreneurship has a significant positive effect on the entrepreneurial subjective norms.
H8	ESN3→ESN:Role models have a significant positive effect on the entrepreneurial subjective norms.
H9	EPBC1→EPBC:Professional ability regarding entrepreneurship has a significant positive effect on the entrepreneurial perceived behavior control.
H10	EPBC2→EPBC:Entrepreneurial ability has a significant positive effect on the entrepreneurial perceived behavior control.
H11	EPBC3→EPBC:Entrepreneurial experiences have a significant positive effect on the entrepreneurial perceived behavior control.
H12	EPBC4→EPBC:Personality traits have a significant positive effect on entrepreneurial perceived behavior control.
H13	ESF→EI: Entrepreneurial situational factors has a significant positive effect on entrepreneurial intention.
H14	ESF→EB: Entrepreneurial situational factors has a significant positive effect on entrepreneurial behavior.
H15	Entrepreneurial implementation intention plays a intervening role between entrepreneurial intention and behavior of college students.

## Materials and Methods

### Participants and Procedures

In this study, the data used for establishing college students’ entrepreneurial intention- behavior extended model were collected with the help of surveys and interviews from students in five colleges located in Wuhu City, Anhui Province, between February and August 2019. Based on the balanced distribution of educational background, gender and age of the students, a questionnaire survey was conducted among the students from 5 colleges in Wuhu, Anhui Province (including liberal arts colleges, science and technology colleges, and junior college, undergraduate and graduate students). Students at these five schools have all received education in innovation and entrepreneurship.

This study distributed 1,186 hard copies of the survey questionnaire and got back 1,013, with 838 effective questionnaires. Among them, the survey data of 175 people were missing, and deletion method was used to delete the missing values. The effective response rate was 82.7%. Among the participating college students, there were 62.2% males, 63.8% between the age range of 20–23 years old, and 67.3% were junior college students. The number of participants from each of the five colleges was comparable, as shown in Table 2.

**TABLE 2:** Sample description.

Category		Frequency	Percentage	Effective percentage
Sex	Male	521	62.2	62.2
	Female	371	37.8	37.8
	Total	838	100.0	100.0
Age	16–19	76	9.1	9.1
	20–23	535	63.8	63.8
	24–27	216	25.8	25.8
	28–30	11	1.3	1.3
	Total	838	100.0	100.0
Education	Junior	564	67.3	67.3
	Undergraduate	216	25.8	25.8
	Graduate	58	6.9	6.9
	Total	838	100.0	100.0
College	Anhui Vocational College Mechanical and Electrical Technology	252	30.1	30.1
	Anhui Engineering University	175	20.9	20.9
	Wuhu Professional Technology College	147	17.5	17.5
	Anhui Normal University	99	11.8	11.8
	Anhui College Traditional Chinese Medicine	165	19.7	19.7
	Total	838	100.0	100.0

In addition to the basic information questionnaire, the Theory of Planned Behavior -based questionnaire that is popular worldwide for investigating entrepreneurial intention studies was used (Hagger et al., 2002). The Theory of Planned Behavior questionnaire included four sub-scales and 15 items. Besides, the validity assessment form of the entrepreneurial behavior questionnaire was sent out to successful entrepreneurs, entrepreneurial mentors, persons in charge of entrepreneurial institutions, graduate representatives who pursued entrepreneurship, and undergraduate representatives, for comments. Based on their feedback, a questionnaire on entrepreneurial behavior was developed in this study. Entrepreneurial Behavior Scale consisted of three dimensions: entrepreneurial situational factors; entrepreneurial implementation intention; and entrepreneurial behavior, and seven questions. Entrepreneurial behavior was the explained variable, and was assigned a value of “1” or “0” when the answer to the question “Have you ever had an entrepreneurial behavior?” was “Yes” or “No,” respectively. Both the entrepreneurial intention scale and entrepreneurial behavior scale were Likert 5-point scale, as shown in Table 3.

**TABLE 3:** Factor analysis, reliability and validity analysis.

Latent variable	Measured dimension	Measurable variables	Factor loading	Number	Cronbach’s alpha	CR	AVE
Y1 Entrepreneurial intention (EI)		EI1: I think I will pursue entrepreneurship in the future. (X1)	0.898	3	0.874	0.777	0.799
		EI2: I think I will pursue entrepreneurship if there is an opportunity. (X2)	0.888				
		EI3: I think I have strong entrepreneurial intentions. (X3)	0.896				
Y2 Entrepreneurial attitude (EA)	Expected results	EA1: I wish I could accumulate money and wealth. (X4)	0.792	5	0.823	0.793	0.586
		EA2: I am longing for social acceptance. (X5)	0.779				
		EA3: I wish I could realize my ideas. (X6)	0.734				
	Perceived utility from expected results	EA4: I wish I could contribute to society and my country. (X7)	0.760				
		EA5: I wish I could have an accomplished career. (X8)	0.762				
Y3 Entrepreneurial subjective norms (ESN)	Reference groups	ESN1: I think successful entrepreneurs affect my choice of entrepreneurship. (X9)	0.832	3	0.779	0.752	0.694
		ESN2: I think my friends and relatives support my entrepreneurship. (X10)	0.821				
		ESN3: I think my teachers and the college support my choice of entrepreneurship. (X11)	0.846				
Y4 Entrepreneurial perceived behavior control (EPBC)	Human capital	EPBC1: I think I have strong professional abilities. (X12)	0.814	4	0.793	0.763	0.617
		EPBC2: I think I am capable of entrepreneurship. (X13)	0.767				
		EPBC3: I think I already have entrepreneurial experiences. (X14)	0.759				
		EPBC4: I think my personality traits are suitable for entrepreneurship. (X15)	0.801				
Y5 Entrepreneurial situational factors (ESF)	Entrepreneurial atmosphere	ESF1: I think the social environment promotes entrepreneurial behavior. (X16)	0.799	3	0.702	0.721	0.627
	Entrepreneurial policies	ESF2: I think government policies promote entrepreneurial behavior. (X17)	0.785				
	Entrepreneurial education	ESF3: I think college education promotes entrepreneurial behavior. I am longing for social acceptance. (X18)	0.791				
Y6 Entrepreneurial implementation intention (EII)	Plan making	EII1: I have made the 1-month plan about when, where, and how to attend entrepreneurial activities. (X19)	0.839	3	0.764	0.768	0.680
	Plan implementation	EII2: I have completed my entrepreneurial activities at a planned location, time, and in the planned way in the past month. (X20)	0.808				
	Realization of the goals	EII3: I achieved my expected goals about attending entrepreneurial activities in the past month. (X21)	0.826				
Y7 Entrepreneurial behavior (EB)		EB1: Do you ever have entrepreneurial behavior? (X22)					

### Statistics

#### Reliability Analysis

In this study, the study used SPSS 24.0 to analyze the reliability of the questionnaire. The overall scale had a Cronbach’s alpha value of 0.946. The six sub-scales had Cronbach’s alpha values greater than 0.7 (range: 0.702–0.874; Table 3). The results demonstrated that the questionnaire had high internal consistency and acceptable reliability.

#### Validity Analysis

This study adapted the questionnaire on the widely used the Theory of Planned Behavior -based entrepreneurial intention instrument. Besides, the validity assessment form of the entrepreneurial behavior questionnaire was sent out to successful entrepreneurs, entrepreneurial mentors, persons in charge of entrepreneurial institutions, graduate representatives who pursued entrepreneurship, and undergraduate representatives, for comments. These measures were taken to ensure the content validity of the questionnaire.

Kaiser-Meyer-Olkin (KMO) test and Bartlett test of sphericity were performed using SPSS 24.0 to determine the construct validity of the questionnaire. The KMO value was 0.968 and was statistically significant (*p* < 0.001). The sample was randomly divided to conduct separate exploratory factor analyses. Exploratory factor analysis provided four factors with eigenvalues greater than 1, and accumulatively explained 72.69 % of the variance. Orthogonal Varimax rotation showed a unidimensional separation on four factors (factor lading range: 0.734–0.898, which were all between 0.5–1.0). These results demonstrated satisfactory construct validity of the questionnaire. Besides, all the composite reliability (CR) values ranged between 0.721 and 0.793 and exceeded the recommended level of 0.7, which demonstrated good composite reliability (CR) of the questionnaire. Average Variance Extracted (AVE) measures the convergent validity. The general criterion is AVE > 0.5. In our study, the AVE ranged between 0.586 and 0.799. The AVE values of all sub-scales exceeded 0.5. The results suggested a good convergent validity of the questionnaire (Table 3).

#### Goodness of Fit Testing of the Structural Equation Model

This study uses the structural equation model (SEM) as a theoretical model to test the proposed extended model and hypothesis of the college students’ entrepreneurial intention-behavior. In the process of hypotheses, satisfactory goodness of fit (GOF) is a necessary condition. Since the samples in this study are not more than 1,000, the study tested the GOF of the SEM equation using maximum likelihood estimation through AMOS 24.0 software. Based on the research opinions of Schreiber, King et al., the following fitting degree indexes were selected. The acceptable standard for χ2/df (0, 5), the acceptable standard for RMSEA was less than 0.09, and the acceptable standards for other indexes were all (0.7, 0.95). All the GOF indices, including absolute fit indices (χ^2^/df and RMSEA, GFI, and AGFI) and incremental fit indices (CFI, NFI, and IFI), met the recommended criteria. As shown in Table 4, the constructed model was acceptable and did not need any further adjustment.

**TABLE 4:** The goodness of fit indices.

The goodness of fit indices	Measured index	Criteria		Goodness of fit
		Acceptable	Good	
Absolute fit indices	χ^2^/df	(0, 5)	<2	1.556
	RMSEA	<0.09	<0.05	0.026
	GFI	(0.7, 0.95)	>0.95	0.967
	AGFI	(0.7, 0.95)	>0.95	0.959
Incremental fit indices	CFI	(0.7, 0.95)	>0.95	0.967
	NFI	(0.7, 0.95)	>0.95	0.914
	IFI	(0.7, 0.95)	>0.95	0.968

#### Results

**FIGURE 3:**
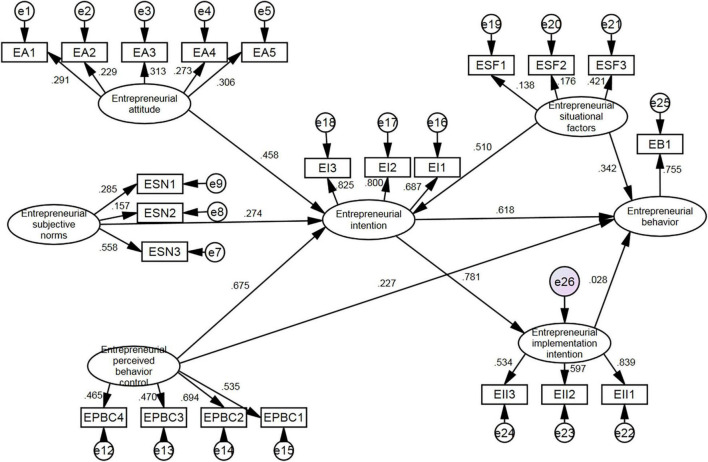
The estimated path coefficient for the college student entrepreneurial intention-behavior extended model.

The path coefficient between latent variables reflects the extent of the cause-effect relationship between the two variables. In this study, AMOS 24.0 software was used to calculate the path coefficients between six variables in the SEM equation including entrepreneurial attitude, subjective norms, perceived behavior control, entrepreneurial intention, entrepreneurial situational factors, and entrepreneurship implementation intention. The path analysis diagram has been presented in Figure 3.

Attainment of material goals, social reputation expectations, individual’s self-evaluation expectation, social responsibility, and career development have significant effects on the entrepreneurial attitude, as revealed by the path coefficients: 0.291, 0.229, 0.313, 0.273, and 0.306, respectively (*p* < 0.001). Therefore, the hypotheses H1, H2, H3, H4, and H5 were accepted. Support from relatives and friends, the college teachers’ views on entrepreneurship and role models have a significant effect on the entrepreneurial subjective norms, as revealed by the path coefficients: 0.285, 0.157, and 0.558, respectively (*p* < 0.001). Therefore, the hypotheses H6, H7 and H8 were accepted. Professional ability, entrepreneurial ability, entrepreneurial experience and personality traits have a significant effect on the entrepreneurial perception behavior control as revealed by the path coefficients: 0.535, 0.694, 0.470, and 0.465, respectively (*p* < 0.001). Therefore, the hypotheses H9, H10, H11, and H12 were accepted. Entrepreneurial situational factors were found to be positively associated with entrepreneurial intention as revealed by the path coefficients: 0.510, respectively (*p* < 0.001). Therefore, the hypothesis H13 was accepted. Entrepreneurial situational factors were found to be positively associated with entrepreneurial behavior, as revealed by the path coefficients: 0.342 (*p* < 0.001) respectively. Therefore, the hypothesis H14 was also accepted. Entrepreneurial implementation intention plays an intervening role between entrepreneurial intention and behavior of college students, as revealed by the path coefficients: 0.781, 0.028, respectively (*p* < 0.001). Therefore, the hypothesis H15 was also accepted.

Moreover, this study further validated the group suitability of the Theory of Planned Behavior -based entrepreneurial intention model in college students. Entrepreneurial attitude, subjective norms and perceived behavior control have a significant positive impact on the entrepreneurial intention as revealed by the path coefficients: 0.458, 0.274, and 0.675, respectively (*p* < 0.001). The results are consistent with the results of previously conducted studies at home and abroad. Meanwhile, this study elucidates the antecedent variables of the formation of undergraduates’ entrepreneurship intention. Entrepreneurial intention and entrepreneurial perceived behavior control have a positive impact on entrepreneurial activities, as revealed by the path coefficients 0.618 (*p* < 0.01) and 0.227 (*p* < 0.01), respectively. The results not only confirm the statement of the Theory of Planned Behavior but also verify that in addition to entrepreneurial intention and entrepreneurial perceived behavior control, entrepreneurial situational factors and entrepreneurial implementation intention are strong supplements for the Theory of Planned Behavior model regarding explanation of behavior. Thus, the college students’ entrepreneurial intention-behavior extended model was validated.

## Discussion

### Path Analysis and Antecedent Variables Analysis for the Initiation of Entrepreneurial Intention

#### Entrepreneurial Attitude and Intention

The results of our empirical study show that three antecedent variables including expectation of material possessions, longing for social reputation and expectation of self-evaluation have a significant positive impact on the entrepreneurial attitude in college students. There are two possible explanations for these results. Firstly, according to the law of somatopsychic development of college students, they choose their career direction at this stage independently. Irrespective of their decision to start entrepreneurship or not, they long for material possessions, people’s recognition and social respect. Secondly, in-person interviews confirmed that the main factors which influence college student’s entrepreneurship include financial rewards, realization of personal ideas and becoming entrepreneurs. The results from the interviews were in conformity with the three predicted antecedent variables.

Two antecedent variables, namely social responsibility and career development, also have a significant positive impact on the entrepreneurial attitude of college students. There are two possible explanations for these results. Firstly, college students have a strong feeling of patriotism and they respond actively to the nation’s call and encouragement for starting new business after completing their graduation. This is reflective of the college students’ strong sense of historical mission and social responsibilities. Secondly, considering the relationship between graduates’ career development and the national economic development, successful entrepreneurship not only energizes the nation’s economic development but also helps an individual progress in his/her career. Therefore, the expectation of entrepreneurship and the perceived value of the expectation of entrepreneurship could significantly affect the entrepreneurial attitude and consequently the intention to pursue entrepreneurship. So, the more positive is the attitude, more will be the efforts made by an individual.

#### Entrepreneurial Subjective Norms and Intention

Among the variables of the Theory of Planned Behavior -based extended model, the reference groups, including students’ relatives and friends, college teachers and entrepreneurial role models, had a significant positive effect on the subjective norms and subsequently on the entrepreneurial intention. There were two possible reasons for this. Firstly, while responding to the innovative entrepreneurial opportunities and education offered by the colleges, the students developed a stronger consciousness of the subjective norms and a greater entrepreneurial intention as the expectation pressures and support from schools and teachers increased. Secondly, successful entrepreneurs, especially the alumni, served as role models and hence provided peer pressure. With greater role model effects and stronger support from families and friends, the college students developed stronger subjective norms consciousness and a greater entrepreneurial intention.

#### Entrepreneurial Perceived Behavior Control and Entrepreneurial Intention

The results of this empirical research revealed that human capital had a positive impact on the college students’ entrepreneurial perceived behavior control. The results also demonstrated that entrepreneurial perceived behavior control is not only subject to change with the graduate’s professional ability, entrepreneurial ability and previous entrepreneurial experiences, but also is affected by the individual’s qualities such as personality traits, independence, self-efficacy, etc. There are several possible reasons for this. Firstly, the accumulation of professional knowledge and skills may help graduates to understand the values of the corresponding sector goods and services better and therefore the foundation for their entrepreneurship is laid. Secondly, the entrepreneurial education in colleges passes the knowledge to students, simulates scenarios of entrepreneurial situations, and trains students with a practical project. Colleges foster students’ entrepreneurial intention and comprehensive ability by using these diversified educational experiences. As a result, students tend to possess a higher entrepreneurial ability and are more likely to identify valuable business opportunities. Thirdly, previous entrepreneurial experience, irrespective of whether it was a success or a failure, tends to inspire entrepreneurship in college students and make them more confident to start actual entrepreneurial activities. Fourthly, college students with extrovert and open-minded personality are not satisfied with a fixed income and a stable life brought by waged employment. They are more willing to take challenges. Entrepreneurship offers higher self-efficacy and self-confidence, which also motivates the college student to become an entrepreneur.

#### Entrepreneurial Situational Factors, Entrepreneurial Implementation Intention, and Entrepreneurial Intention

Research results showed that entrepreneurial situational factors and implementation intentions correlated positively with the college student’s entrepreneurial intention. Entrepreneurship support policies, entrepreneurship education and social entrepreneurship atmosphere will directly influence students’ entrepreneurial intentions, especially in China. Before the formation of entrepreneurial implementation intention, the entrepreneurial intention determines whether the students will participate in the entrepreneurial activities or not. However, in some particular entrepreneurial situational factors, some students may form certain entrepreneurial implementation intentions and habits, which indirectly boost their entrepreneurial intention. The results show that entrepreneurial situational factors and entrepreneurial implementation intention correlated positively with the college student’s entrepreneurial intention. They also indicate that, for some college students, beginning of entrepreneurship is the result of the external entrepreneurial environment and unconscious implementation of entrepreneurial intentions.

To sum up, it can be said that, the hypotheses proposed in the entrepreneurial intention-behavior model were accepted which confirmed the antecedent variables and paths of entrepreneurial intention formation. The empirical study also revealed that the three factors, entrepreneurial attitude, entrepreneurial subjective norms, and entrepreneurial perceived behavior control correlated positively with entrepreneurial intention and the correlation was significant. The result is consistent with the results of previous researches conducted at home and abroad. At the same time, the research proves that the entrepreneurial situational factors significantly affect the entrepreneurial intention of college students, especially in China.

### Analysis of Influencing Factors of Entrepreneurial Behavior

#### Entrepreneurial Situational Factors and Entrepreneurial Behavior

Although Fishbein and Ajzen (2010) believe that the entrepreneurial situational factors can only indirectly affect the entrepreneurial intention and entrepreneurial behavior, the empirical research shows that the entrepreneurial situational factors are correlated positively with entrepreneurial behavior. There can be several possible reasons for it. Firstly, today’s society encourages and advocates entrepreneurship from the perspectives of public opinion, publicity and values, and respects the college students’ choice of starting their own business. As a result, college students become more motivated to turn their entrepreneurial intention into actual behavior. Secondly, both the state and local government have formulated policies to encourage college students to start their own business, provide financial support, tax incentives, business incubation platform, and training to them. These policies and measures facilitate the realization of a college graduate’s dream of entrepreneurship and help in successful transition into an entrepreneur. The responses made by the graduates during interviews revealed that the entrepreneurial policies had a significant positive impact on their entrepreneurial behavior. Thus, it implies that the entrepreneurial policies effectively promote the initiation of actual entrepreneurial activities in the students who possess entrepreneurial intentions. Last but not the least, on the basis of the surveys and interviews, it was found that all the colleges offered various kinds of entrepreneurial education and training, which provided convenient venues, courses and training to improve students’ entrepreneurial skills and helped in smooth transition of their entrepreneurial intention into entrepreneurial behavior. The college students benefit a lot from such practices. These results once again demonstrate that guidelines laid down by the Chinese government to promote entrepreneurship in college students are foresighted and meaningful.

#### Entrepreneurial Implementation Intention, Entrepreneurial Intention, and Entrepreneurial Behavior

An implementation intention is a combination of setting a goal, situation, and goal-directed actions. To be specific, the subject is inclined to participate in entrepreneurial activities and modifies his/her behavior to accommodate a particular future situation (when, where, and how) to automatize goal attainment. The research showed that implementation intention increases the likelihood of exhibiting actual entrepreneurial behavior. College students with implementation intentions are more likely to conquer the obstacles, automatize the implementation intentions, strengthen their entrepreneurial intention, and consequently initiate and maintain their entrepreneurial behavior. This happens because the undergraduates frequently participate in innovative entrepreneurial courses, entrepreneurial training, and various entrepreneurial competitions. Although participating in such entrepreneurial activities are initiatives taken by the school and are not fully reflective of the students’ entrepreneurial intentions, but it is believed that such situational factors promote the automatization of implementation intention and help in modification of habit and behavior. In other words, active participation in entrepreneurship-related future-planning activities and adopting an entrepreneurial behavior unconsciously in such situations tends to foster inculcation of entrepreneurial intention and promotes initiation of entrepreneurial behavior (Li, 2014).

To sum up, the study introduced entrepreneurial situational factors and entrepreneurial implementation intention into the basic the Theory of Planned Behavior framework, constructed the extended model and validated the suitability of our extended model in college student’s entrepreneurial intention-behavior research. It has been proved by empirical studies that entrepreneurial situational factors can directly have a significant impact on entrepreneurial intention and entrepreneurial behavior, especially in China. Entrepreneurial implementation intention plays an intervening role between entrepreneurial intention and behavior of college students. The study also proved the inclusion of entrepreneurial situational factors and entrepreneurial implementation intention in the model is a beneficial supplement to the basic the Theory of Planned Behavior model.

### Limitations

This study has several limitations. First, this study adopted the Theory of Planned Behavior as the theoretical framework introduced entrepreneurial situational factors and entrepreneurial implementation intention, and constructed a two-step extended entrepreneurial intention—behavior model. Due to the limited objectives, some potential regulating variables were not included in the extended model, such as entrepreneurial resilience and entrepreneurial emotion and other personal traits. Future research can explore more factors that can influence the transformation from entrepreneurial intention to entrepreneurial behavior of college students by combining quantitative analysis. Secondly, the range of subjects for the empirical study needs to be widened. For example, only ungraduated college students were included in this study. However, it was felt that, the alumni who have graduated in the past 5 years could have been included, so as to diversify the sample, making it more heterogeneous. Thirdly, more appropriate methods can be adopted for study in future. This study used surveys and interviews. In the future, the study could consider to follow up the entrepreneurial intention and behavior in the participants to further validate our extended model regarding its prediction efficacy about the entrepreneurial intention and behavior, in particular, tracking and analyzing the internal mechanism of entrepreneurial implementation intention can be further carried out in the future.

## Conclusion

Currently, the academic circle has systematically studied the influencing factors of college students’ entrepreneurial intention based on the Theory of Planned Behavior. However, the influencing factors of how college students with strong entrepreneurial intention turn into entrepreneurs are limited, and the existing theoretical model of planned behavior cannot explain this transformation mechanism.

This study adopted the Theory of Planned Behavior as the theoretical framework introduced entrepreneurial situational factors and entrepreneurial implementation intention, and constructed a two-step extended entrepreneurial intention—behavior model. The structural equation was constructed using AMOS24.0 to empirically analyze the antecedent variables of college students’ entrepreneurial intention and the factors influencing entrepreneurial behavior. It further expands the explanatory ability of the Theory of Planned Behavior in the field of college students’ entrepreneurial behavior.

During the entrepreneurial intention formation period, entrepreneurial attitude, entrepreneurial subjective norms, entrepreneurial perceived behavior control and entrepreneurial situational factors had a direct effect on the entrepreneurial intention. The antecedent variables of entrepreneurial attitude include expected material possessions, expected social status, expected self-evaluation, responsibility, mission and career development. The entrepreneurial subjective norms are affected by the college students’ families and friends, the college teachers and role models. Entrepreneurial perceived behavior control is affected by the students’ professional ability, entrepreneurial ability, entrepreneurial experiences and personality traits. This study analyzes in detail the path of the first stage of the expansion model affecting the entrepreneurial intention of college students and the internal relationship among the influencing factors, and explains the contradictions between previous research conclusions.

During the transition to actual entrepreneurial actions, the situational factors such as entrepreneurial social atmosphere, government policies and the entrepreneurial education provided by the colleges played an important role. These measures regulate an individual’s entrepreneurial intention through creation of entrepreneurial activity plans, performing entrepreneurial activities, realization of the associated goals, etc. Entrepreneurial implementation intention plays an intervening role between entrepreneurial intention and behavior of college students. For the second phase of development model not only makes up the gap between the previous research on entrepreneurial intention and behavior transformation, but also provides some theoretical reference and basis for further revealing the path of entrepreneurial intention from behavior regulation. So, to sum up, the empirical study offers a fresh perspective on the Theory of Planned Behavior -based entrepreneurial intention-behavior research.

## Data Availability Statement

The raw data supporting the conclusions of this article will be made available by the authors, without undue reservation.

## Ethics Statement

The studies involving human participants were reviewed and approved by Ethics Review Committee of Anhui Vocational College of Mechanical and Electrical Technology. Written informed consent to participate in this study was provided by the participants’ legal guardian/next of kin.

## Author Contributions

DL conceived the research idea, designed the experiment, performed the analyses, and drafted the manuscript.

## Conflict of Interest

The author declares that the research was conducted in the absence of any commercial or financial relationships that could be construed as a potential conflict of interest.

## Publisher’s Note

All claims expressed in this article are solely those of the authors and do not necessarily represent those of their affiliated organizations, or those of the publisher, the editors and the reviewers. Any product that may be evaluated in this article, or claim that may be made by its manufacturer, is not guaranteed or endorsed by the publisher.
